# Characterization, epidemiology and risk factors of multiple drug allergy syndrome and multiple drug intolerance syndrome: A systematic review

**DOI:** 10.1002/clt2.12190

**Published:** 2022-08-22

**Authors:** Parbir K. Jagpal, Saad Alshareef, John F. Marriott, Mamidipudi Thirumala Krishna

**Affiliations:** ^1^ Institute of Clinical Sciences University of Birmingham Birmingham UK; ^2^ University Hospitals Birmingham NHS Foundation Trust Birmingham UK; ^3^ Institute of Immunology and Immunotherapy University of Birmingham Birmingham UK

**Keywords:** multiple drug allergy, multiple drug allergy syndrome, multiple drug hypersensitivity, multiple drug intolerance, multiple drug intolerance syndrome

## Abstract

**Background:**

Multiple drug allergy and multiple drug intolerance syndrome (MDAS/MDIS) labels are an impediment to clinical care and knowledge regarding these conditions is limited. This systematic review investigated the characterization, epidemiology, risk factors, clinical impact and pharmaco‐economics of MDAS and MDIS.

**Methods:**

Systematic literature search across 11 databases (01 January 2000–06 November 2020) for MDIS, MDAS and related terminology. Studies were reviewed for quality of evidence and risk of bias by employing Critical Appraisal Skills Programme cohort study checklist. A narrative synthesis approach facilitated by systematic textual descriptions, tabulation and thematic analysis was adopted.

**Results:**

There was heterogeneity in terminology and methodology. Few studies applied standard drug allergy diagnostic methods. There is some evidence to suggest that multiple drug hypersensitivity syndrome (MDHS; i.e., confirmed allergies in MDAS) is a distinct clinical entity. Prevalence of MDIS and MDAS labels in unselected & selected populations varied between 2.1%–6.4% & 4.9%–90% and 1.2% & 0%–36% respectively. Reported risk factors included female gender, increasing age, body mass index, anxiety, depression, co‐morbidities, concurrent allergies and increased healthcare utilization. Drugs commonly implicated were antibiotics and non‐steroidal anti‐inflammatory drugs. No studies relating to clinical impact and pharmaco‐economics were found.

**Conclusion:**

There is considerable burden of MDAS and MDIS labels. Data needs cautious interpretation as majority of studies described involved unverified labels. Despite this limitation and heterogeneity of studies, there is some evidence to suggest that MDHS is a distinct clinical entity. Well‐designed multi‐centre studies applying standardized terminology and diagnostic methodology are needed to gain further insight into these conditions.

## 1 INTRODUCTION

Adverse drug reactions (ADRs) are a response to a medicine that is noxious and unintended^1^ and are broadly classified into two types. Type A reactions are an exaggerated response to a drug's normal pharmacological action when administered at the standard therapeutic dose.^1^, ^2^ Type B reactions are unpredictable responses based on known pharmacological actions of the drug.^1^, ^3^ Both reactions are dose‐independent. Type B reactions however, can be potentially life‐threatening and may warrant change in treatment.

Drug allergy (type B ADR), is a terminology that is employed in the context of a ‘true’ hypersensitivity reaction (HSR) as per Gell and Coombs classification and is usually a Type‐1 (immediate or IgE mediated) or Type‐4 (non‐immediate or T cell mediated) HSR.^2^, ^3^, ^4^, ^5^ Skin tests are useful in the investigation of Type‐1 and Type‐4 HSRs.^6^

Drug intolerance is not immunologically mediated and may be pseudo‐allergic or idiosyncratic.^2^, ^3^ A drug reaction is less likely to have an allergic basis in the absence of histamine‐mediated symptoms or systemic involvement, and if it is characterized by non‐specific symptoms or if isolated gastrointestinal symptoms are reported.^4^, ^5^ Mechanisms underpinning drug intolerance are poorly understood.^2^, ^5^ It is the least specific term for an ADR and may be added into a health record to avoid subsequent use of a drug.

Drug intolerances are commonly mislabelled as an ‘allergy’ in patient records. Inaccurate drug allergy labelling has been extensively studied in high income countries (HICs) in the context of penicillin allergy labels. Between 90% and 95% of penicillin allergy labels are inaccurate, leading to prescription of expensive broad‐spectrum antibiotics which enhance risk of antimicrobial resistance, *Clostridioides difficile* infection, surgical site infections, lengthen hospital stay and increase healthcare costs.^7^, ^8^, ^9^ Reported penicillin allergy, with or without multiple drug intolerance (MDI) syndrome has been shown to increase healthcare utilization with an increase in number of visits per follow‐up.^8^ Poor documentation and knowledge gaps amongst healthcare professionals have been linked to inaccurate penicillin allergy labelling.^10^, ^11^, ^12^, ^13^

Multiple drug allergy syndrome (MDAS) refers to patients describing symptoms suggestive of a HSR to ≥1 drug class. Multiple drug intolerance syndrome (MDIS) on the other hand refers to patients describing ADRs suggestive of a non‐immunological reaction to ≥3 drug classes. Given the unmet need of specialist allergy services globally, limitations and onerous nature of drug allergy tests, MDAS and MDIS labels are an impediment to healthcare delivery, particularly in the context of antimicrobial stewardship.^14^, ^15^

The main aim of this study was to systematically review published evidence to:

- determine prevalence and risk factors for MDAS and MDIS
- characterize MDAS and MDIS
- determine the clinical impact and pharmaco‐economics of MDAS and MDIS.

## 2 METHODS

A systematic literature search was conducted across 11 data bases (MEDLINE, EMBASE, PsycINFO, Web of Science [Core Collection], CINAHL plus [EBSCO], Cochrane Library [Wiley], Scopus [ELSEVIER], PubMed [USNLM], NICE Evidence, PROQUEST, LexisNexis) from 01 January 2000 to 06 November 2020, with no language restrictions. Key words included Multiple drug allergy (MDA) OR Multiple drug allergy syndrome (MDAS) OR MDI OR MDIS OR Multiple drug hypersensitivity (MDH) and MESH terms included: (epidemiology OR cohort stud* OR cohort analys* OR cross‐sectional stud* OR cross sectional analys* OR observational analys* OR prevalence OR disease frequency OR incidence OR rate). Search terms were agreed and refined by reviewers (PJ, SA, JM, TK) after an initial scoping exercise.

The systematic review protocol was registered with PROSPERO (CRD CRD42022302225), an international prospective register of systematic reviews based at the University of York Centre for Reviews and Dissemination.^16^ Whilst the primary aim of our systematic review was to investigate MDAS and MDIS, this study also included closely related conditions as identified in the literature search including MDH, multiple drug hypersensitivity syndrome (MDHS), MDI, multiple antibiotic sensitivity syndrome (MASS) and polyallergy (PA). Abstract only publications, conference presentations, letters, grey literature, reviews, and meta‐analyses were excluded. The report was structured using the Preferred Reporting Items for Systematic Reviews (PRISMA).^17^

A total of 10,728 records across all databases were exported to the reference management tool ‘Endnote’. Removal of duplicates resulted in 7041 title and abstract records being screened by the first reviewer (PJ) who then applied the exclusion criteria to remove 7023 records. The second reviewer (SA) reviewed 10% of the excluded records and there was consensus for the exclusions. Eighteen records were assessed for eligibility. One further study was identified for review from bibliographies. Full text review of 19 studies was carried out independently by two reviewers (PJ and SA). Third (TK) and fourth (JM) reviewers provided a consensus opinion with agreement that the 19 studies were suitable for the systematic review.

**FIGURE 1:**
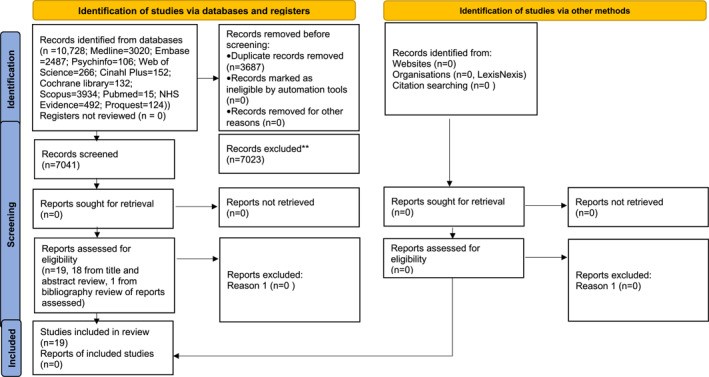
PRISMA 2020 flow diagram for new systematic reviews: searches of databases, registers and other sources, exclusion and included studies. ** exclusion criteria: abstract only publications, conference presentations, letters, grey literature, reviews, and meta‐analyses

Figure 1: shows the PRISMA^18^ flow chart.

Studies were reviewed for quality of evidence and risk of bias by applying the Critical Appraisal Skills Programme cohort study checklist.^18^ This method was chosen due to methodological heterogeneity of the studies reviewed. A systematic narrative synthesis facilitated by systematic textual descriptions, tabulation and thematic analysis was adopted due to the heterogeneity of studies.

Quality assessment of basic drug allergy work up was conducted by comparing to British and European guidelines.^4^, ^5^, ^6^, ^19^, ^20^, ^21^, ^22^, ^23^, ^24^, ^25^ Standards of diagnostic methodology were assessed against a checklist of parameters:

- Clinical history
- Clinical examination
- Acute and baseline serum total tryptase
- Skin tests (skin prick tests, intradermal tests) ± serum specific IgE ± patch tests for Type I and IV HSR
- Drug provocation test (DPT or drug challenge test).

## 3 RESULTS (Tables 1, 2, 3)

### 3.1 Definitions and diagnosis

The systematic review revealed multiple nomenclature in the context of patients presenting with an allergy or intolerance to multiple drugs. This was based on the number of drugs involved, whether they were different drugs or from unrelated drug classes and if the patient was ‘truly’ allergic based on a systematic assessment involving a clinical history, allergy testing and/or a DPT when deemed appropriate. Table 1 lists acronyms along with respective definitions used in previous studies.

**TABLE 1:** Definitions used in studies included in systematic review

Components of definition	Definition with reference to drug classes/unrelated drugs^a^					
	MDAS^26^, ^27^, ^28^, ^29^	MDIS^26^, ^30^, ^31^, ^32^, ^33^	MDI^20^, ^34^	MDH^35^, ^36^, ^37^, ^38^, ^39^	MDHS^40^	MASS^41^
Reactions to different drug classes	√					
Reactions to ˃1 different drug class	√					√
Reactions ≥2 different drug classes	√			√	√	
Reactions ≥2 different drug classes, immunologically mediated	√			√		
Reactions ≥3 different drug classes		√	√			
Reactions ≥3 different drug classes on 3 different occasions, not immunologically mediated		√	√			
Diagnostic methodology						
Clinical history	√	√	√	√	√	√
Clinical examination		√				
Serum tryptase (2 samples)		√				
Skin tests (prick and intradermal)		√		√	√	
Patch tests		√		√	√	
DPTs	√	√		√	√	
Serum specific Ig E	√	√		√		

Abbreviations: DPT, drug provocation test; MASS, multiple antibiotic sensitivity syndrome; MDAS, multiple drug allergy syndrome; MDH, multiple drug hypersensitivity; MDHS, multiple drug hypersensitivity syndrome; MDI, multiple drug intolerance; MDIS, multiple drug intolerance syndrome; PA, polyallergy.

^a^ Reference made to ‘drug classes/unrelated drugs’, excluding references to ‘drugs’ included in MDIS2^5^ and PA^42^.

MDAS was referred to as a *reaction* to ˃1 different drug class in one study^26^ and as an *allergy* to ≥2 in one study.^30^ One study did not specify number of drug classes and used the term ‘multiple drug intolerance’ interchangeably with MDAS.^31^ MDIS referred to as ADR/HSR/intolerance to ≥3 drug classes in five studies,^27^, ^30^, ^32^, ^40^, ^42^ and to ≥3 drugs by Omer et al.^25^ MDI was referred to as ADR/intolerance to ≥3 drug classes in two studies.^28^, ^42^ MDH or MDHS was referred as HSR/allergy to ≥2 drug classes in six studies.^29^, ^33^, ^34^, ^35^, ^36^, ^41^ MASS was referred to as sensitivity to ˃1 drug class in one study.^37^ PA was referred to as an ADR to ≥3 drugs in a single study.^38^

### 3.2 Countries, setting, design, population type and sample size

The majority of studies (*n* = 14) were carried out in HICs including UK (2),^20^, ^25^ Italy (5),^28^, ^29^, ^31^, ^32^, ^39^ USA (3),^26^, ^30^, ^42^ Switzerland (1),^38^ France (2),^36^, ^40^ and Canada (1),^41^; three from upper middle‐income countries including Serbia,^35^ Turkey,^37^ and South Africa^33^ and two from low middle‐income countries including Nigeria,^34^ and India.^27^ HIC studies tended to be in secondary care and allergy units^20^, ^25^, ^26^, ^28^, ^29^, ^31^, ^32^, ^36^, ^38^, ^39^, ^40^, ^41^ or across primary and secondary care.^30^, ^42^ There was considerable variation in study design, sample size, and clinical setting and 13 out of 19 (68%) studies involved retrospective analysis. These characteristics are summarised in Table 2.

**TABLE 2:** Key characteristics of 19 studies included in systematic review

Author, year and country	Prospective/Retrospective (P/R) R C ‐(review of patient records only for specified condition)	Primary/Secondary care (P/S)	Cohort study or non‐cohort (C/NC);Sample size (*N* = ) suspected allergy, ADR, HSR population/unselected population	Mean age (years) (±SD)/reported age information	Condition	Definition used for MDA/MDAS/MDI/MDIS/MDH/MDHS/MASS/PA	% of patients diagnosed with respective condition from specified cohort	Risk factors identified	Penicillin allergy/intolerance/hypersensitivity/sensitivity implicated (Yes/No, comments)
MDAS									
Nettis et al., 2001^29^ Italy	R	S	C *N* = 460 suspected allergy population	42 ± 18	MDAS	MDAS is characterized by reactions to ˃1 different class of antibiotics	23% of suspected allergy population	For MDAS: Female sex Intolerance to NSAIDs For positive tolerance test: Male sex Intolerance to NSAIDs History of MDAS	Yes Penicillin allergy in 46% of patients 45% of patients sensitive to one drug class only were sensitive to penicillins; 51% of patients sensitive to one or more drug classes were sensitive to penicillins
Ramam et al., 2010^27^ India	R	S	C *N* = 23 suspected ADR population	36.4 ± 12.4	MDAS	Multiple drug hypersensitivity (MDHS)/MDAS drug allergies to ≥2 structurally or pharmacologically unrelated drugs/drug classes	0% of suspected allergy population	For MDAS: Female sex	No
Asero et el, 2002^28^ Italy	P	S	C *N* = 120 (study 1), *N* = 261 (study 2) suspected allergy population	39 (study 1) 42 (study 2)	MDAS	MDAS is reaction against different, chemically unrelated antibiotic or non‐antibiotic drugs/drug classes	30% MDAS antibiotics 36% MDAS NSAIDS of suspected allergy population	MDAS Female sex H/O multiple intolerance to antibiotics risk factor for multiple tolerance to NSAIDs H/O intolerance to NSAIDs is risk factor for multiple intolerance to Abx History of multiple drug intolerance was a risk factor for intolerance to an alternative, chemically unrelated drug	No
MDIS									
Schiavino et al., 2007^32^ Italy	R	S	C *N* = 480 suspected ADR population	17–83	MDIS	MDIS is HSR to ≥3 drugs that are chemically, pharmacologically, & immunogenically unrelated/drug classes, taken on 3 different occasions and with negative allergy test reactions	90% of suspected ADR population	For MDIS: Female sex Increasing age Family history of atopy	No
De Pasquale et al., 2012^31^ Italy	P	S	C *N* = 30 suspected ADR population	46.87 (±9.80)	MDIS	MDIS is non‐allergic drug HSR to ≥3 drugs, chemically, pharmacologically & immunogenically unrelated/drug classes, manifested upon 3 different occasions, and with negative allergy testing	Not reported	Anxiety Depression Alexythimia Somatisation of symptoms	No
Macy et al., 2012^30^ USA	R R C	P & S	C *N* = 2,375,424 unselected population	62.4 ± 16.1	MDIS	MDIS is defined as intolerance to ≥3 unrelated drug classes	2.1% of unselected population	Female sex Increasing age Increasing BMI Increased healthcare utilization Higher medication usage Higher incidence of new allergy Increased medical attention sought for common non‐morbid conditions Anxiety	Yes Penicillin allergy in 7.85% of patients with history of allergy to at least one drug class New penicillin allergy in 0.51% of patients with no history of allergy at start of study period
Omer et al., 2014^25^ UK	R R C	S	C *N* = 25,695 suspected allergy population	60	MDIS	MDIS is ADR to ≥3 drugs without a known immunological mechanism	4.9% of suspected allergy population	For MDIS: Female sex Multiple co‐morbidities Previous hospital admissions Allergies to broad spectrum of drugs including non‐penicillin antibiotics (exception of penicillin)	Yes Penicillin allergy in 53% of patients
Peter, 2016^33^ South Africa	R	S	NC *N* = 1 suspected allergy population	38	MDIS	MDIS is ADR to ≥3 unrelated drugs/drug classes	100% of suspected ADR population	For MDIS: Female sex	Yes Single patient study, penicillin allergy present
MDAS & MDIS									
Blumenthal et al., 2018^26^ USA	R R C	S	C *N* = 746,888 unselected population	MDIS median 57 (inter‐quartile range 45–68) MDAS median 52 (inter‐quartile range 41–63)	MDIS MDAS	MDIS intolerances to ≥3 drug classes, MDAS is HSR to ≥2 drug classes with a possible immunologic mechanism.	6.4 (MDIS) 1.2 (MDAS) 0.4 (both MDIS & MDAS) of unselected population	Increasing age with MDIS and MDAS Female sex with MDIS and MDAS White ethnicity with MDIS and MDAS Anxiety & depression with MDIS Odds ratio for anxiety or depression greater with number of drug class intolerances (MDIS) Other allergies with MDIS Smoking with MDIS Alcohol use with MDIS Co‐morbidities with MDIS Frequent inpatient and emergency room use with MDAS Depression with MDAS Chronic urticaria/angioedema with MDAS Frequent outpatient utilization with MDAS	Yes Penicillin intolerance in 41.7% MDIS patients Penicillin allergy in 50.8% MDAS patients
MDI									
Antoniou et al., 2016^20^ UK	R R C	S	C *N* = 55 suspected ADR population	66 (±9)	MDI	MDI is ADR to ≥3 unrelated drug classes	10% of suspected ADR population	Increasing age Female sex White European ethnicity Anxiety disorder Gastroesophageal reflux disease	No
Okeahialam, 2017^34^ Nigeria	R R C	P	C *N* = 489 unselected population	Range from 38 to 71	MDI	MDI is intolerance to ≥3 different drug classes with no clear immunological mechanism	3.1% of unselected population	For MDI: Female sex Increasing age Anxiety Depression	No
MDH									
Gex‐Collet et al., 2005^38^ Switzerland	P	S	C *N* = 7 suspected allergy population	20–80	MDH	MDH is drug allergy to ≥2 chemically different drugs/drug classes	100% of suspected allergy population Two types of MDH reported: Simultaneous (3 pts)/sequential (4 pts) administration	Severe drug allergy may predispose to development of second drug allergy	Yes Penicillin hypersensitivity in 71% of patients
Columbo et el, 2009^39^ Italy	P	S	C *N* = 120 suspected ADR population	52.2	MDH	MDH is HSR to ≥2 drugs with different molecular structure/drug classes	23.3% of suspected ADR population	Female sex Auto‐immune thyroiditis	Yes Penicillin hypersensitivity in 29% of patients
Atanaskovic‐Markovic et al., 2012^35^ Serbia	P	S	C *N* = 279 suspected HSR population	2–14	MDH	MDH relates to ≥2 more chemically different drugs/drug classes	2.5% of suspected HSR population Two types of MDH reported: Simultaneous (2 pts)/sequential (5 pts) administration	‐Female sex	Yes Penicillin hypersensitivity in 29% of patients
Studer et al., 2012^36^ France	R	S	C *N* = 1925 suspected ADR population	28–79	MDH	MDH is sensitisation to ≥2 chemically unrelated substances/drug classes,	0.6% of suspected ADR population	For MDIS: Female sex Increasing age	Yes Penicillin hypersensitivity in 43% of patients
Guvenir et al., 2019^37^ Turkey	P	S	C *N* = 73 suspected HSR population	6–10	MDH	MDH is immunologically‐mediated HSR ≥2 chemically different drugs/drug classes	2.7% of suspected HSR population	Not identified	Yes Penicillin hypersensitivity in 100% of patients
MDHS									
Landry et al., 2020^40^ France	R	S	C *N* = 9250 suspected allergy population	41.6 (range,16–80)	MDHS	MDHS is HSR to ≥2 chemically and pharmacologically unrelated drug/drug classes	2.5% of suspected allergy population	Female sex	Yes Penicillin hypersensitivity in 71% of patients
POLYALLERGY									
Jimenez et al., 2019^42^ USA	R R C	P & S	C *N* = 2,007,434 unselected population	Poly‐allergy – 50.4 ± 13.5 Ultra‐poly‐allergy – 52.4 ± 13.0	PA	Polyallergy (5–9 drugs) ultra‐poly‐allergy (≥10 drugs) documented drug ADRs regardless (If medications are related)	1.7% of unselected population	‐Increasing age Female sex Increased healthcare utilization Increased use of psychotropic medication Increased mental health disorders Increased functional somatic syndrome	No
MASS									
Park et al., 2000^41^ Canada	R R C	S	C *N* = 850 suspected ADR population	26.1 ± 26.3 months	MASS	MASS is antibiotic sensitivity to ˃1 class of antibiotic	11% of suspected ADR population	For MASS Female sex History of atopy (eczema or asthma) Family history of adverse drug reactions to antibiotics	Yes Penicillin sensitivity in 86% of patients

Abbreviations: MASS, multiple antibiotic sensitivity syndrome; MDAS, multiple drug allergy syndrome; MDH, multiple drug hypersensitivity; MDHS, multiple drug hypersensitivity syndrome; MDI, multiple drug intolerance; MDIS, multiple drug intolerance syndrome; PA, polyallergy.

The study population varied. Five cohort studies included suspected allergy patients (MDAS,^28^, ^29^ MDIS,^25^ MDH,^38^ MDHS^40^); seven included suspected ADR patients (MDAS,^27^ MDIS,^29^, ^31^ MDI,^20^ MDH,^36^, ^39^ MASS^41^); two included suspected HSR patients (MDH^35^, ^37^) and all were in secondary care. Four were in unselected populations (MDAS and MDIS,^26^ MDIS,^30^ MDI,^34^ PA^42^) of which two were across both primary and secondary care (MDIS,^30^ PA^42^), one in primary care (MDI^34^) and one in secondary care (MDAS and MDIS^26^). One MDIS study was a single case of a suspected allergy patient in secondary care.^33^

Fourteen studies included adult populations (>18 years) only^20^, ^25^, ^26^, ^28^, ^29^, ^30^, ^31^, ^33^, ^34^, ^36^, ^38^, ^39^, ^40^, ^42^ with an age range of 18–80,^38^ mean age (standard deviation) reported were between 46.87 (±9.80)^31^ to 66 ± 9 years.^20^

Three studies included children only^35^, ^37^, ^41^ with an age range of 2–14 years, mean age (standard deviation) reported in one study was 26.1 ± 26.3 months.^41^ Two reported age ranges of 2–14 years^35^ and 6–10 years.^37^ Two studies included adults and children^27^, ^32^ with a child age range of 14^27^–17 years.^32^ One reported age range only of 17–8332^26^ and one reported mean age (standard deviations) as 36.4 ± 12.4 years.^27^

### 3.3 Diagnosis of respective condition

The proportion of patients diagnosed with MDAS, MDIS and other related conditions in unselected and selected (i.e. those with a suspected allergy/ADR/HSR) populations in different settings showed variation. MDAS diagnosis was reported in a secondary care unselected population as 1.2%^26^ and ranged from 0%,^42^ 23%,^29^ to 36%^28^ in suspected allergy/ADR populations. Similarly, MDIS diagnosis ranged from 2.1%^30^ across primary & secondary care in an unselected population, 6.4%^26^ in an unselected population in a secondary care setting, 4.9% in a suspected allergy population^25^ and 90%^32^ in a suspected ADR population. Both MDAS and MDIS were reported by Macy et al.^30^ as 0.4% in an unselected population in secondary care. MDI diagnosis was reported in primary care as 3.1% in an unselected population^34^ and 10% in a suspected ADR population in secondary care.^20^ MDH diagnosis was reported in secondary care selected populations ranging from 0.6% (suspected ADR^36^) 2.5%^35^ and 2.7%,^37^ (suspected HSR) and 23.3%^39^(suspected ADR) to all seven patients in a small suspected allergy cohort.^38^ MDHS diagnosis in a larger secondary care suspected allergy population was reported as 2.5%.^41^

All selected populations were in secondary care (MDAS,^28^, ^29^, ^42^ MDIS,^25^, ^31^, ^32^, ^33^ MDIS,^20^ MDH,^31^, ^35^, ^36^, ^37^, ^38^ MDHS,^40^ MASS^41^) and employed more than one diagnostic methodology.^27^, ^28^, ^29^, ^31^, ^32^, ^33^, ^35^, ^36^, ^37^, ^38^, ^39^, ^40^ Unselected populations were in primary care (MDI),^34^ secondary care (MDAS, MDIS)^26^ and across both (MDIS,^30^ PA^42^) and used clinical history only.

Two studies reported two types of MDH^35^, ^38^: (a) developing to different drug classes administered ‘simultaneously’ (i.e., during the same episode), and (b) developing to different drug classes administered ‘sequentially’ (i.e. occurring at separate episodes in a given patient). One study reported that three patients developed MDH simultaneously and four patients sequentially,^38^ the other study reported that two patients developed MDH simultaneously and five patients sequentially.^35^ Reactions included severe cutaneous adverse reactions (SCARS) and drug reaction with eosinophilia and systemic symptoms (DRESS) syndrome. MDHS was reported as 2.5% in a suspected allergy population,^40^ PA as 1.7% of an unselected population^42^ and MASS as 11% of a suspected ADR population.^41^

### 3.4 Common drugs implicated

A variety of drugs were implicated, but most common were antibiotics and non‐steroidal anti‐inflammatory drugs (NSAIDs). Whilst some studies focussed on specific drug groups only (anti‐hypertensives^20^, ^34^ and antibiotics^29^, ^41^), the majority found a range of drugs to be involved including anti‐epileptics, opioids, angiotensin converting enzyme inhibitors, corticosteroids and psychotropics. 12 out of 19 studies (63%) implicated penicillin allergy.^25^, ^26^, ^29^, ^30^, ^33^, ^35^, ^36^, ^37^, ^38^, ^39^, ^40^, ^41^

Studies with larger patient numbers (>250 patients)^26^, ^30^, ^36^, ^37^, ^38^, ^39^, ^41^, ^42^ and those including drug allergy workup^26^, ^29^, ^33^, ^36^, ^37^, ^41^ identified greater numbers of drug classes. These were a mixture of retrospective reviews of patient records and prospective studies, the majority were carried out in HICs.

### 3.5 Risk factors

Risk factors for MDI/MDIS/MDA/MDAS/MDH/MASS/PA were reported in a number of studies. Female sex in 16 studies^20^, ^25^, ^26^, ^27^, ^28^, ^29^, ^30^, ^32^, ^33^, ^34^, ^35^, ^36^, ^39^, ^40^, ^41^, ^42^ and increasing age^20^, ^26^, ^30^, ^32^, ^34^, ^36^, ^42^ in seven studies were most frequently reported. White European ethnicity was identified as a risk factor in two studies for MDAS^26^ and MDI^20^ although this may be reflective of the ethnicity of the population studied (UK,^20^ USA^26^). A large study in the UK involving electronic in‐patient records of a 25,695 multi‐ethnic population performed univariate and multivariate analyses and found no statistically significant association between age, ethnicity or weight and MDIS.^25^ One large study from USA reviewing records of 2,375,424 patients found increasing body mass index (BMI)^30^ to be a risk factor in contrast to the UK study.^25^ Mental health disorders were reported as a risk factor for MDIS,^42^ anxiety was identified as a risk factor for MDI^20^, ^34^ and MDIS,^30^, ^31^ and depression as a risk factor for MDI,^34^ MDIS^31^ and MDAS.^26^ Anxiety and depression was a risk factor for MDIS and more likely with increased number of drug intolerances.^26^ Alexythima (difficulty in taking part in social situations or maintaining relationships) was identified as a risk factor in a small study of 30 MDIS patients.^31^

Other risk factors for MDAS included chronic urticaria or angioedema and frequent in‐patient and emergency room visits.^26^ A history of multiple antibiotic intolerance was a risk factor for multiple NSAID intolerance and vice versa.^28^ Intolerance to NSAIDs was a risk factor for MDAS.^31^ Risk factors for MDIS included smoking and alcohol consumption,^26^ family history of atopy,^32^ somatisation of symptoms,^31^, ^42^ increased use of psychotropic medication^42^ and co‐morbidities^25^, ^26^ Associations were reported with increased healthcare utilization, emergency room and outpatient attendance and previous hospital admissions for MDIS.^25^, ^30^, ^42^ A study of 2,375,424 patient medical notes reported that MDIS patients were more likely to seek medical attention for common non‐morbid conditions and had increased medication usage^30^ Three studies identified current allergies as a risk factor for MDIS^25^, ^26^, ^30^ although one did not find prior allergy to penicillin to be a risk factor.^25^ Risk factors for MDH included current allergies^38^ and auto‐immune thyroiditis.^39^ A history of eczema or asthma and family history of ADRs to antibiotics were reported as a risk factors for MASS.^41^

### 3.6 Quality assessment of studies

Comparison of quality of studies was challenging due to variation in study design with respect to clinical setting, cohort size and characteristics, definition and diagnostic approach and whether specialist drug assessment was conducted. There was also risk of referral bias by patient or clinician particularly in secondary care settings and allergy clinics.^20^, ^25^, ^26^, ^27^, ^28^, ^29^, ^30^, ^31^, ^32^, ^33^, ^35^, ^36^, ^37^, ^38^, ^39^, ^40^, ^41^, ^42^

Larger cohort studies in unselected populations in USA (*N* = 746,888^26^; *N* = 2,007,434^42^; *N* = 2,375,424^30^) did not refer to guidelines or use diagnostic methodology, relying on history taking, patient recall, and/or retrospective review of records, thus risking potentially poor data quality related to limitations of patient recall and/or inaccurate record keeping. Studies including additional confirmation of diagnosis^27^, ^28^, ^29^, ^31^, ^32^, ^33^, ^35^, ^36^, ^37^, ^38^, ^39^, ^40^ were more likely to generate reliable datasets, although sample size varied from a single case study^33^ to 9250.^40^ Smaller cohort sizes (<100 patients)^20^, ^27^, ^31^, ^33^, ^37^, ^38^, ^39^, ^41^ do not support generalizability of findings. Table 3 summarises the quality assessment of studies.

**TABLE 3:** Quality assessment of drug allergy workup and studies included in systematic review

Author, year and country	Quality of basic diagnostic methodology as per international guidelines^4^, ^5^, ^6^, ^19^, ^20^, ^21^, ^22^, ^23^, ^24^, ^25^ (Yes/No): Clinical history Clinical examination Serum tryptase (2 samples) Skin tests (prick and intradermal) Patch tests DPTs, Serum Ig E	Patients characterized as per current international guidelines (Yes/HSR not investigated/confirmed)	Quality assessment and limitations of study (use of the Critical Appraisal Skills Programme (CASP)^18^ cohort study checklist)
MDAS			
Nettis et al., 2001^29^ Italy	**Clinical history** √ Clinical examination x Serum tryptase (2 samples) x Skin tests (prick and intradermal) x Patch tests x **DPTs** √ **Serum specific Ig E** √	No HSR not investigated/confirmed	Well‐designed, well documented data from patient records, detailed clinical history, all patients subject to oral challenges
Ramam et al., 2010^27^ India	**Clinical history** √ Clinical examination x Serum tryptase (2 samples) x Skin tests (prick and intradermal) x Patch tests x **DPTs** √ Serum specific Ig E x	No HSR not investigated/confirmed	Small patient number (23)
Asero et el, 2002^28^ Italy	**Clinical history** √ Clinical examination x Serum tryptase (2 samples) x Skin tests (prick and intradermal) x Patch tests x **DPTs** √ Serum specific Ig E x	No HSR not investigated/confirmed	No epidemiological basis, H/O multiple allergy may increase self‐referral and referral by clinicians
MDIS			
Schiavino et al., 2007^32^ Italy	**Clinical history** √ Clinical examination x Serum tryptase (2 samples) x **Skin tests (prick and intradermal)** √ **Patch tests** √ **DPTs** √ **Serum specific Ig E** √	**Yes** HSR not investigated/confirmed	Use of pre‐medication (sodium cromolyn or oral antihistamines) may have reduced reactions and affected identification of intolerance
De Pasquale et al., 2012^31^ Italy	**Clinical history** √ **Clinical examination** √ Serum tryptase (2 samples) x **Skin tests (prick and intradermal)** √ **Patch tests** √ DPTs x **Serum specific Ig E** √	**Yes** HSR not investigated/confirmed	Small number of patients (30) Female patients only
Macy et al., 2012^30^ USA	**Clinical history** √ Clinical examination x Serum tryptase (2 samples) x Skin tests (prick and intradermal) Patch tests x DPTs x Serum specific Ig E x	No HSR not investigated/confirmed	No allergy workup Retrospective data extraction from patient records, documentation may be poor/inaccurate
Omer et al., 2014^25^ UK	**Clinical history** √ Clinical examination x Serum tryptase (2 samples) x Skin tests (prick and intradermal) x Patch tests x DPTs x Serum specific Ig E x	No HSR not investigated/confirmed	No allergy workup Retrospective data extraction from patient records, documentation may be poor/inaccurate
Peter, 2016^33^ South Africa	**Clinical history** √ **Clinical examination** √ **Serum tryptase (2 samples)** √ **Skin tests (prick and intradermal)** √ Patch tests x DPTs x Serum specific Ig E x	No HSR not investigated/confirmed	Single case study
MDAS & MDIS			
Blumenthal et al., 2018^26^ USA	**Clinical history** √ Clinical examination x Serum tryptase (2 samples) x Skin tests (prick and intradermal) x Patch tests x DPTs x Serum specific Ig E x	No HSR not investigated/confirmed	No allergy workup Retrospective data extraction from patient records, documentation may be poor/inaccurate
MDI			
Antoniou et al., 2016^20^ UK	**Clinical history** √ Clinical examination x Serum tryptase (2 samples) x Skin tests (prick and intradermal) x Patch tests x DPTs x Serum specific Ig E x	No HSR not investigated/confirmed	No allergy workup Risk of referral bias from practitioners and self‐referral from patients more engaged in their care Retrospective data extraction from patient records, documentation may be poor/inaccurate Small number (5) identified as MDI‐anti‐hypertensives
Okeahialam, 2017^34^ Nigeria	**Clinical history** √ Clinical examination x Serum tryptase (2 samples) x Skin tests (prick and intradermal) x Patch tests x DPTs x Serum specific Ig E x	No HSR not investigated/confirmed	Number of patients (489) No allergy workup Retrospective data extraction from patient records, documentation may be poor/inaccurate
MDH			
Gex‐Collet et al., 2005^38^ Switzerland	**Clinical history** √ Clinical examination x Serum tryptase (2 samples) x Skin tests (prick and intradermal) **Patch tests** √ DPTs Serum specific Ig E x	No HSR not investigated/confirmed	Small number of patients (7) Tests performed at least 6 weeks after patients recovered from allergic reactions, some >10 years after first reaction, skin or LTT often positive years after the allergic reaction
Columbo et el, 2009^39^ Italy	**Clinical history** √ Clinical examination x Serum tryptase (2 samples) x Skin tests (prick and intradermal) Patch tests x **DPTs** √ Serum specific Ig E x	No HSR not investigated/confirmed	Small number of patients (28)
Atanaskovic‐Markovic et al., 2012^35^ Serbia	**Clinical history** √ Clinical examination x Serum tryptase (2 samples) x **Skin tests (prick and intradermal)** √ **Patch tests** √ **DPTs** √ **Serum specific Ig E** √	**Yes definition for positive prick & intradermal skin tests stated, not for immediate or delayed HSR; reports 33 immediate,** **180 delayed, 66 both types of reactions in separate episodes**	Children only. Small number (7/279) identified as MDH
Studer et al., 2012^36^ France	**Clinical history** √ Clinical examination x Serum tryptase (2 samples) x **Skin tests (prick and intradermal)** √ **Patch tests** √ **DPTs** √ Serum specific Ig E x	**Yes** HSR not investigated/confirmed	Small patient number (11/1925 identified as MDH)
Guvenir et al., 2019^37^ Turkey	**Clinical history** √ Clinical examination x Serum tryptase (2 samples) x **Skin tests (prick and intradermal)** √ Patch tests x **DPTs** √ Serum specific Ig E x	**Yes definition for immediate and delayed HSR when history taking stated,** **Definition for positive prick & intradermal skin tests stated;** **Confirmed HSR in 7 patients** **Immediate only (*n* = 3), both (*n* = 4)**	Number of patients (73)
MDHS			
Landry et al., 2020^40^ France	**Clinical history** √ Clinical examination x Serum tryptase (2 samples) x **Skin tests (prick and intradermal)** √ **Patch tests** √ **DPTs** √ Serum specific Ig E x	**Yes definition for immediate and delayed HSR stated;** **59 positive skin/patch testing; 21 immediate: 38 delayed. 33 positive DPTs: 19 immediate, 14 delayed**	Not all patients with alleged drug hypersensitivity were tested as only drugs used in patient's care were reviewed
POLYALLERGY			
Jimenez et al., 2019^42^ USA	**Clinical history** √ Clinical examination x Serum tryptase (2 samples) x Skin tests (prick and intradermal) x Patch tests x DPTs x Serum specific Ig E x	No HSR not investigated/confirmed	No allergy workup Retrospective data extraction from patient records, documentation may be poor/inaccurate
MASS			
Park et al., 2000^41^ Canada	**Clinical history** √ Clinical examination x Serum tryptase (2 samples) x Skin tests (prick and intradermal) x Patch tests x DPTs x Serum specific Ig E x	No HSR not investigated/confirmed	Telephone calls and questionnaires to parents not children Recall bias as many events occurred earlier than clinic visit, accuracy of parent recollections time of ADR may be affected Referral bias into allergy clinic

Abbreviations: DPT, drug provocation test; LTT, lymphocyte transfer tests; MASS, multiple antibiotic sensitivity syndrome; MDAS, multiple drug allergy syndrome; MDH, multiple drug hypersensitivity; MDHS, multiple drug hypersensitivity syndrome; MDI, multiple drug intolerance; MDIS, multiple drug intolerance syndrome; PA, polyallergy.

## 4 DISCUSSION

This is the first comprehensive systematic review evaluating the characterization, epidemiology and risk factors of MDAS and MDIS and related conditions. This review included 18 cohort studies and one case study and majority of research was conducted in HICs. This review identified multiple nomenclature (and acronyms) for patients presenting with suspected allergies and intolerance to multiple drugs. MDAS was reported as 1.2% in an unselected population^26^ and ranged from 0%,^27^ to 23%,^29^ and 36%^28^ in suspected drug allergy/ADR cohorts. Similarly, MDIS ranged from 2.1%^40^ to 6.4%^30^ in unselected populations, and 4.9% in a suspected drug allergy cohort,^39^ and 90%^42^ in a suspected ADR cohort. Similarly, the diagnosis of MDH was reported ranging from 0.6% in a suspected ADR cohort,^35^ 2.5%^33^ and 2.7%,^34^ in suspected HSR cohorts, 23.3% in a suspected ADR^29^ cohort. MDHS diagnosis in a larger study involving a suspected drug allergy population was reported at 2.5%.^41^ This systematic review did not identify studies investigating the impact of these conditions on clinical outcomes or pharmaco‐economics.

There was a notable variation in definitions used across studies for various conditions referring to an allergy or intolerance to multiple drugs with respect to the number of drugs/drug classes implicated and application of standard diagnostic methodology, thereby not allowing meaningful comparisons. The indiscriminate use of the word ‘allergy’ as an umbrella term to cover all ADRs has become a major barrier in routine clinical practice, particularly during management of infections. Some studies noted that documentation of allergies in electronic health records (EHRs) may be inaccurate due to the use of ‘allergy’ as a generic term to include HSRs, intolerances, drug toxicity, idiosyncratic reactions and other ADRs.^25^, ^26^, ^30^, ^42^ This highlights the need for standardized definition and terminology, robust education for all prescribers (including trainees and students) and appropriate fit for purpose, equitable and standardized IT systems within health services. Recommendation of standardized terminologies was not within the scope of this review but is an area for further research.

Standard diagnostic methodology and reference to British and European guidelines was employed in labelling patients in six studies.^31^, ^32^, ^35^, ^36^, ^37^, ^40^ This included clinical history, clinical examination, serum specific IgE, skin tests (skin prick test/intradermal test and/or patch test) ± DPT^4^, ^19^, ^21^, ^22^, ^23^, ^24^, ^43^, ^44^ and followed European guidance (European Network for Drug Allergy, European Academy of Allergy and Clinical Immunology). These studies involved diagnostic labels of MDH,^35^, ^36^, ^37^ MDHS,^40^ and MDIS^31^, ^32^ and systematically evaluated patients to confirm a diagnosis of an immunologically‐mediated reaction.

The most commonly implicated drugs were antibiotics and NSAIDs. Penicillin allergy was implicated in 12 out of 19 studies (63%).^26^, ^30^, ^33^, ^35^, ^36^, ^37^, ^38^, ^40^, ^41^ Studies with larger sample sizes (>250 patients)^25^, ^26^, ^30^, ^32^, ^35^, ^36^, ^40^, ^42^ and those that included a drug allergy workup^31^, ^32^, ^35^, ^36^, ^37^, ^40^ identified a greater number of drug classes.

The most frequently reported risk factors were female

sex^20^, ^25^, ^26^, ^27^, ^28^, ^29^, ^30^, ^32^, ^33^, ^34^, ^35^, ^36^, ^39^, ^40^, ^41^, ^42^; age^20^, ^26^, ^32^, ^34^, ^36^, ^42^; increased healthcare utilization^25^, ^26^, ^30^, ^42^; mental health disorders^42^ including anxiety and depression^20^, ^26^, ^30^, ^31^, ^34^; and presence of co‐morbidities.^25^, ^26^ Whilst White European ethnicity was identified as a risk factor in two studies^20^, ^26^ this was not confirmed in another study.^25^ One study found increasing BMI to be a risk factor,^30^ although a further study found no such association.^25^

Other risk factors included smoking, alcohol, chronic urticaria or angioedema^26^; eczema, asthma^41^; family history of atopy^32^ and family history of ADRs to antibiotics^41^; seeking medical attention for common non‐morbid conditions,^30^ somatisation of symptoms^31^, ^42^; increased use of psychotropic medication,^42^ increased medication usage^30^; auto‐immune thyroiditis^39^; concurrent allergies,^25^, ^26^, ^28^, ^30^, ^38^ history of MDI as a risk factor for multiple NSAID intolerance and history of NSAID intolerance a risk factor for multiple antibiotic intolerance.^28^

Studies involving MDHS^40^ and some involving MDH^35^, ^37^ confirmed an underlying HSR, thereby supporting the notion that these are distinct clinical entities. Whilst the true prevalence of MDH and MDHS has not yet been established, current data suggests some heterogeneity. Three patterns have been reported including those with an immediate HSR to multiple drug classes, non‐immediate HSR to multiple drug classes and a mixed pattern of immediate and non‐immediate HSR to multiple drug classes.^35^, ^37^, ^40^ Furthermore, ‘simultaneous’ (during the same episode) and ‘sequential’ (during separate occasions) MDH in the context of SCARS and DRESS syndrome has also been reported.^35^, ^38^ There is also some evidence for a role for persistent T‐cell activation involving a subset of CD4^+^ CD25^dim^, CD38^+^, and PD‐1^+^ T cells in MDHS.^45^, ^46^, ^47^ It is however unclear if MDIS is a distinct clinical syndrome, as it is a clinical diagnosis based on subjective and varied symptomatology without an immunological basis and with no confirmatory in vivo or in vitro tests.

This systematic review process was robust, addressed the study research aims and adhered to PRISMA guidelines.^48^ The review spanned over 2 decades with no language limitations and used wide search terms. There were however multiple limitations in published evidence including heterogeneity in nomenclature, definitions and terminology employed, clinical settings (primary or secondary care), bias towards HICs, retrospective nature of some studies with wide variation in sample sizes with some being relatively small and a number of studies reported prevalence based on unverified labels.

Whilst there is no published evidence regarding the impact of MDAS and MDIS labels (and related conditions) on clinical care and pharmaco‐economics, experience from inaccurate penicillin allergy labels in HICs suggests a significant impact on clinical outcomes, healthcare utilization and healthcare costs.^8^, ^49^, ^50^, ^51^, ^52^, ^53^ Alongside provision of education in basic aspects of drug allergy labelling/de‐labelling, there is a real need to standardize international nomenclature and diagnostic criteria for patients reporting an allergy or intolerance to multiple drugs, as no International Statistical Classification of Diseases codes currently exist.^54^ There is scope for further research into MDH/MDHS, in particular to identify risk factors including possible human leucocyte antigen (HLA)^40^ associations via a pharmacogenomics approach.

A multi‐pronged approach is needed focussing on development of standardized international nomenclature, education and training of healthcare professionals to facilitate standardized methods for accurate documentation alongside establishment of referral pathways for drug allergy testing.

Guyer et al.^55^ highlighted the adverse clinical impact of indiscriminate and inaccurate use of the term ‘allergy’ in EHRs. MDAS and MDIS patients should undergo specialist allergist evaluation involving systematic clinical history, review of previous clinical records, investigations including skin tests, and supervised single/graded drug challenge procedures (with or without placebo) for verification of status followed by appropriate amendment of EHR and clear communication to both patient and family physician regarding their up to date ‘allergy’ status.^55^ Including additional fields in EHRs to capture drug interactions, idiosyncratic responses, metabolic/disease‐specific intolerance etc., might provide useful information to discriminate between immune and nonimmune mediated ADRs.^55^

Prospective real time capture of data in all clinical settings employing a standardized electronic platform might enable generation of accurate clinical datasets regarding ADRs. This approach in conjunction with robust clinical pathways for drug allergy testing, needs to be considered in shaping policies giving due consideration regarding unmet demand of allergy specialists and variations in health service frameworks.

In conclusion, published literature suggests that there is a considerable burden of MDAS and MDIS labels and related conditions, particularly in HICs. There is some evidence to suggest that MDH and MDHS are distinct clinical entities as studies involving MDHS and some involving MDH confirmed an underlying HSR.

## AUTHOR CONTRIBUTIONS

**Parbir Kaur Jagpal**: Conceptualization (Lead); Data curation (Lead); Formal analysis (Lead); Investigation (Lead); Methodology (Lead); Project administration (Lead); Resources (Lead); Validation (Lead); Visualization (Lead); Writing – original draft (Lead); Writing – review & editing (Lead). **Saad Alshareef**: Data curation (Supporting); Formal analysis (Supporting); Writing – review & editing (Supporting). **John F. Marriott**: Conceptualization (Supporting); Formal analysis (Supporting); Methodology (Supporting); Supervision (Lead); Validation (Supporting); Visualization (Supporting); Writing – review & editing (Supporting). **Mamidipudi Thirumala Krishna**: Conceptualization (Supporting); Formal analysis (Supporting); Methodology (Supporting); Supervision (Equal); Validation (Supporting); Visualization (Supporting); Writing – review & editing (Supporting).

## CONFLICT OF INTEREST

Mamidipudi Thirumala Krishna's department received educational grants from ALK Abello, Allergy Therapeutics, MEDA and other pharmaceutical companies for annual PracticAllergy course. Mamidipudi Thirumala Krishna has received grants from NIHR, MRC CiC, GCRF and FSA outside of the work presented in this manuscript. Other authors have none to declare.
